# Evaluation of the Cytotoxic and Apoptogenic Effects of Glabridin and Its Effect on Cytotoxicity and Apoptosis Induced by Doxorubicin Toward Cancerous Cells

**DOI:** 10.15171/apb.2019.057

**Published:** 2019-08-01

**Authors:** Masoud Modarresi, Marziyeh Hajialyani, Narges Moasefi, Farahnaz Ahmadi, Leila Hosseinzadeh

**Affiliations:** ^1^Pharmaceutical Sciences Research Center, Health Institute, Kermanshah University of Medical Sciences, Kermanshah, 6734667149, Iran.; ^2^Medical Biology Research Center , Health Institute, Kermanshah University of Medical Sciences, Kermanshah, Iran.

**Keywords:** Apoptosis, Cytotoxicity, Doxorubicin, Glabridin, Herbal medicine, Licorice

## Abstract

***Purposes:*** In the present study, we tried for the first time to examine the anti-proliferative and anti-apoptogenic effect of Glabridin (Glab) toward three groups of cancer cells (SKNMC, H1299, and A2780). Furthermore, the possibility of co-administration of Glab with doxorubicin (DOX) to these cells was also examined to find out whether Glab can potentiate the cytotoxic effect of this chemotherapy agent.

***Methods:*** Different cellular assays (MTT, caspase-3 activity, MMP, RT-PCR analysis) were carried out on the cancer cells treated with Glab.

***Results:*** Cellular toxicity assay revealed that Glab can potentially reduce the viability of these cells with IC50 concentrations up to 10, 12, and 38 μM toward A2780, SKNMC, and H1299 cell lines, respectively. The results of MMP and caspase-3 activity assays, in association with the results corresponding to the BAX and Bcl-2 gene expressions, altogether revealed that Glab can exert apoptogenic effect on these cells. The intrinsic mitochondrial pathway was found to be the main mechanism, in which Glab induced apoptosis toward H1299 cells and SKNMC cells, while the apoptosis mechanism for A2780 cells could be probably through extrinsic pathway. Glab also potentiated the cytotoxic effect of DOX and its accumulation in H1299 cell line.

***Conclusion:*** The results of this study revealed the promising cytotoxic role of Glab on different carcinoma cells. These data also suggested that co-chemotherapy method using Glab could be effective for treatment of cancer, but further in-vivo and clinical studies are still needed to assure these results.

## Introduction

Cancer could be taken into account as one of the major leading causes of human death across the world. According to the reports, this fatal disease causes mortality of more than 13 million in 2030.^1^ The eradication of cancer in a long-term program has long been investigated by scientists. Several common medications have been developed for prevention and treatment of cancers, but these methods are mostly associated with various drawbacks such as off-target toxic effects, low accumulation in the targeted cells, high costs, and various side effects.^2^ These drawbacks strongly motivated scientists to identify effective methods for cancer prevention. Chemoprevention is one of the promising methods utilizing the medicaments capable in suppression and postponing tumorigenesis. Natural products have long been used for prevention and/or treatment of different cancers.^3,4^ Phytochemicals, mostly used as dietary supplements, possess antioxidant and antineoplastic activities, enhance cellular defense, and possess the capability to reverse or postpone tumorigenesis, due to presence of biologically activate constituents.^5^ Duo to these advantages and limited side effects of phytochemicals, administration of these compounds as substitute and/or complementary chemo-preventive agents could probably create a great success. Licorice (*Glycyrrhiza glabra*, liquorice) is one of the most frequently used herbs which has been performed as a life-enhancing herb from 2100 BC.^6^ Various pharmacological properties are reported for this plant. The antioxidant, anti-inflammatory, detoxification, antiviral, anti-carcinogenic, anti-mutagenesis, tumor suppression, gastro-protective, neuro-protective, anti-diabetic, and immune-modulatory effects of licorice are only some of the pharmacological effects of this herb, which made it as a well-known medicinal herb frequently used for treatment of a wide range of diseases.^7-12^ Glabridin (Glab) is a prenylated isoflavone isolated from the root of licorice. As one of the major constituents of licorice, Glab exerts several biological activities such as antioxidant, antibacterial, anti-atherogenic, anti-osteoporosis, estrogenic, and neuroprotective.^13-16^ Apart from these activities, the anti-cancer and chemoprevention effects of Glab have been reported in the literature.^13,17^ It could also enhance the efficacy of different anti-cancer therapeutics and affect the chemotherapy by inhibiting the synthesis of P-glycoprotein (multidrug resistance protein 1).^18^ In the current study, the cytotoxic effect of Glab was evaluated on SKNMC, H1299, and A2780 carcinoma cell lines. This study reveals the effect of Glab on these cell lines and the mechanism in which Glab inhibits proliferation of cancer cells. The possible effect of Glab on toxicity of doxorubicin (DOX) against these cells and the resistance of cells to this drug has also been investigated in the present study.

## Materials and Methods

Methanol, ethyl acetate, n-hexane, and HPLC-grade methanol were purchased from Merck, (Germany). Deuterated chloroform (CDCI3, 99.8% D atom) was purchased from Acros Organics (United States). Sep-Pak Silica^®^ cartridge (10 g/60 mL) was prepared from Waters (United States). PerfectSil Target ODS-3^®^HPLC column (250×4.6 mm, 5 µm) and VertiSep^®^GES C-18 HPLC column (250×21 mm, 10 µm) were from Knauer (Germany) and Vertical (Thailand), respectively. Dimethyl sulphoxide (DMSO), 2′,7′-dichlorofluorescin diacetate (DCF-DA), 3-(4,5-dimethylthiazol-2-yl)-2,5-diphenyltetrazolium bromide (MTT), DOX, Triton X-100, and rhodamine 123 were supplied from Sigma-Aldrich (St Louis, MO, USA). Trypsin-EDTA was procured from Bon Yakhteh (Iran). Fetal bovine serum (FBS) was purchased from Gabon (United States).

### Preparation of plant material

The *G. glabra* roots were harvested from West-Islamabad (Kermanshah province, Iran) in November 2016. The plant was authenticated in Herbarium of the Ferdowsi University of Mashhad (Mashhad, Razavi-Khorasan province, Iran) and a voucher specimen (with voucher number of 25947) was deposited in the herbarium.

### Extraction and Purification of Glab

In this study, firstly the crud extract was prepared using maceration method. For this purpose, 500 mL ethyl acetate was added to 50 g of the dried powder of *G. glabra* root and stirred for 24 h. The extract was filtrated and filtrate was collected. The extraction was repeated two times on the residue with the same conditions. The final extract was dried using rotary evaporator (Heidolph, Germany) under a reduced pressure condition and at low temperature (≤ 40°C). The obtained crude extract was subjected to solid phase extraction (SPE) on silica gel cartridge (Sep-Pak Silica^®^ cartridge, 10 g/60 mL). In order to isolate different fractions of the crud extract, 2 g of the dried extract was weighed and added to 4 mL of ethyl acetate-hexane (with 1:4 volume ratio). After conditioning the cartridge with 150 mL n-hexane and 150 mL ethyl acetate-hexane (with 1:4 volume ratio), 4 mL of extract solution was loaded on cartridge and consecutively rinsed with a mixture of ethyl acetate and n-hexane with different volume ratios (1:4, 1:2, 1:1, and 1:0). Then the isolated fractions were collected and each fraction was dried at temperature below 40°C using a vacuum-rotary evaporator. Thereafter, the dried fractions were dissolved in methanol and filtered through a membrane filter (0.22 µm pore size) and then subjected separately to HPLC (Knauer, Germany) to analyze the components and recognize the Glab*-*riched fraction (the fraction which contains the highest percentage of Glab). HPLC analysis was conducted using the solvent system of water: methanol according to the method reported in our previous study on a C18 column (250×4.6 mm, 5 μm).^16^ Detection was carried out using UV detector on 225 nm (Detector 2600 version 7605). The Glab*-*riched fraction was subjected again to HPLC and Glab was purified by reversed-phase preparative HPLC and its purity was assessed by analytical HPLC, according to our previous study.^16^

### MTT assay

#### Cell culture conditions

In this study, SKNMC (Human neuroblastoma cells), A2780 (human ovarian carcinoma cell line), and H1299 (human non-small cell lung carcinoma cells) were supplied from Pasteur Institute of Iran (Tehran, Iran). Theses cancerous cell lines were incubated in Dulbecco’s modified Eagle’s medium (DMEM-F12) supplemented with inactivated fetal bovine serum (10% v/v) and 1% penicillin/streptomycin (100 U/mL: 100 U/mL) for cell grows at 37°C in a humidified incubator containing 5% CO_2_. Cell culture medium was replenished at predetermined time intervals (2-3 days) until the cell proliferation had reached 70%–80% confluence. Then the cells sub-cultured and seeded at appropriate population density.

### The effect of Glab on the proliferation of cancerous cell lines

MTT assay was used to examine the effect of Glab on proliferation of different cancerous cell lines. In order to assess the cytotoxic effect of Glab on H1299, SKNMC, and A2780, cells were seeded separately in a 96-well culture plate while the same density of 2× 10^4^ cell/well. A stock solution of Glab was prepared in DMSO and serial dilution was carried out to prepare different concentrations of Glab (2.5-150 μM). For this assay, a group of untreated cells were performed as control, and other groups were treated with different concentrations of Glab. At appropriate time intervals, medium was replenished with 0.5 mg/mL MTT solution and plates were further incubated for 3 h at 37^°^C. After the incubation period, and to solubilize the formazan crystals, 100 µL DMSO was added to each well. The optical density at 570 nm (OD570) was detected (reference wavelength 630nm) using an Eliza micro plate reader (BioTek Instruments, USA) and cell viability percentage was determined. The IC_50_ value was obtained, which was corresponding to the concentration required for killing 50% of cells. All the MTT assays were conducted in triplicate.

### The effect of Glab on caspase-3 activity

Caspase-3 activity was determined using sigma colorimetric caspase kit. For this purpose, cancer cells were seeded in a 96-well plate and incubated for 24 h. The IC_50_ concentration of Glab was performed in this assay. The IC_50_ concentration of Glab was added to wells and incubated for the next 24 h. A group of cells (control) received DMSO and had not received Glab. Thereafter, the treated cells were centrifuged at 1200 rpm for 5 min and were lysed in 15 μL of cell lysis buffer included with the kit. The extraction of the protein content of cells was achieved by centrifugation of lysates at 16 000-20 000 rpm and 4°C for 15 min. The substrate reaction buffer containing caspase-3 substrate was added to the supernatant and incubated for 2 h at 37°C. The absorbance was then measured at 405 nm using a plate reader (BioTek, H1M).

### Measurement of mitochondrial membrane potential

The mitochondrial membrane potential (MMP) was assessed using Rhodamin123, as fluorescent dye. For this purpose, different cancerous cell lines (H1299, SKNMC, and A2780) were seeded separately in 6-well tissue culture plates and incubated for 24 h. After the incubation period, the IC_50_ concentration of Glab was added to the wells and incubated for 24 h. At the end of treatment, 15 µL rhodamine 123 (4 µM) was added to wells and plate was incubated for 30 minutes at 37°C. Thereafter, cells were lysed with 1ml Triton-X100 and kept at 4^°^C for 30 minutes. Then cells were centrifuged at 13 000 rpm and their fluorescence was measured at exciting wavelength of 488 nm and emission wavelength of 590 using fluorescence microplate reader (BioTek, H1M). The protein content of samples was also determined using Bradford assay.^19^

### Real time RT-PCR analysis

The total RNA of H1299, SKNMC, and A2780 cancer cells were extracted using the RNA isolation kit (Roche, Mannheim, Germany) and were assessed qualitatively and quantitatively by spectrophotometer (NanoDrop 2000, USA). Then samples were kept at -80°C for further investigations. Quantitative specific RNA expression was performed in one step express one-step SYBR R Green ER™ kit (Invitrogen, cat #11780–200). The primers used in this study were selected from previously published studies.^20^ Thermal cycler conditions were 15 min at 50°C for cDNA synthesis, 10 minutes at 95°C followed by 40 cycles of 15 seconds at 95°C to denature DNA and 45 seconds at 60°C to anneal and extend the template. All the experiments were carried out in triplicate using a Corbett system (Australia). Expression of target genes was normalized against β-actin. ΔΔCT method was used to measure fold increase of genes in comparison to control group.

### The effect of Glab on the DOX-induced cytotoxicity in cancerous cell lines

In addition to the effect of Glab on the proliferation of the performed cell lines, the effect of this herbal compound on the cytotoxicity induced by DOX on cancer cells (H1299, SKNMC, and A2780) were investigated. DOX is a potent anticancer agent, which has been frequently used for treatment of different cancers. The aim of this section is to identify whether Glab can potentiate the cytotoxic effect of DOX against these cancer cells (H1299, SKNMC, and A2780). For this purpose, in addition to the control group (untreated cells), a group of cells were only treated with DOX (with different concentrations in the range of 3.4-25.5 μM), to be compared with the corresponding Glab-treated ones. The Glab*-*treated cells were prepared by co-incubation of different concentrations of Glab (i.e., 5, 10, and 15 μM) and DOX (3.4-25.5 μM) with cancer cells for 24 h. The viability percentage of cells of each group and their corresponding IC_50_ was measured and compared.

### Effect of Glab on the accumulation of DOX in the cancerous cell lines

In order to identify the effect of Glab on accumulation of DOX in H1299 cell line, cells were incubated with different concentrations of Glab (15 and 30 μM) and DOX for 2 h and then were lysed with Triton-X100 and the amount of their fluorescence was measured at exciting wavelength of 480 nm and emission wavelength of 590 nm using fluorescence microplate reader (BioTek, H1M). It should be worth-noting that the amount of protein in each sample was measured using Bradford assay.

### Statistical analysis

In the present study, all the experiments were conducted in triplicate and reported values represented as the mean value ± SEM. The One-way analysis of variance (ANOVA) using Tukey’s test was performed to compare the results. The statistical significance of variations could be confirmed at P-values less than 0.05.

## Results

### Cytotoxicity effect of Glab on cancerous cell lines

**Figure 1:**
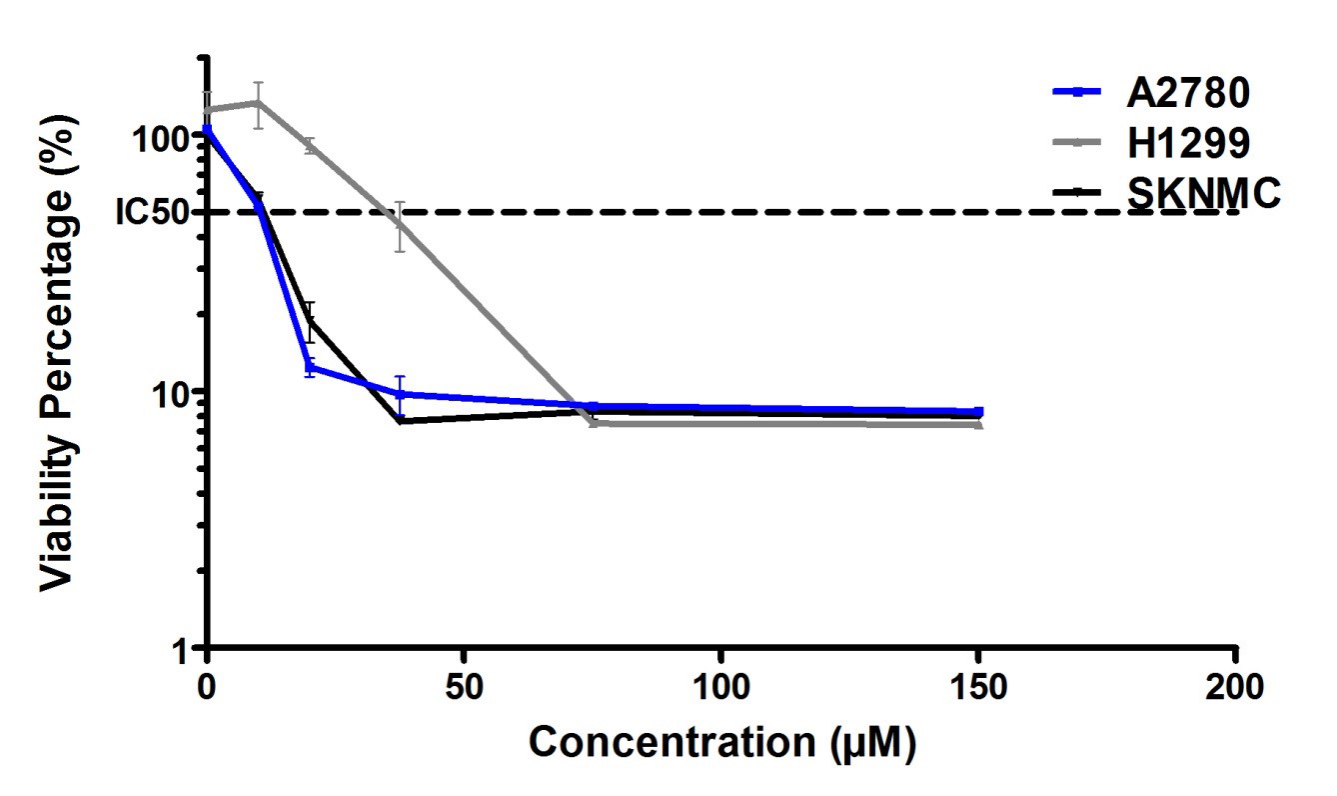
The cytotoxic effect of Glab toward different cancerous cell lines. The cell viability was determined by MTT assay after 24 h exposure as described in materials and methods. Data are expressed as the mean ± SEM of three separate experiments.

The cell viability assay was used to determine the viability of cancerous cells (H1299, SKNMC, and A2780) after exposure to different concentrations of Glab, and to investigate whether treatment of cells with these concentrations of Glab could reduce the viability of these cells. Figure 1 shows the percentage of viable cells after exposure to different concentrations of Glab, in comparison to the control group (100% viability). A glance at this figure reveals that this phytochemical can successfully inhibit the proliferation of all the three cancerous cell lines. For the case of H1299, exposure of Glab with concentrations lower than 25 μM caused no significant effect on the cancerous cells, and Glab was effective on these cells at concentrations higher than 25 μM. Furthermore, the cytotoxic potential of Glab toward these cancerous cells was not dose-dependent at concentrations higher than 75 μM. The viability percentage of these three groups of cancerous cell lines reached a plateau at the concentration of 75 μM, suggesting that Glab is effective on these cells at doses up to 75 μM. This herbal compound was found potent in killing up to 90% of the cancerous cells, for all the three cell lines. The IC_50_ values of Glab toward these cells were separately calculated, and it was 10, 12, and 38 μM toward A2780, SKNMC, and H1299 cell lines, respectively.

### Effect of Glab on the caspase-3 activity of the cancerous cells

**Figure 2:**
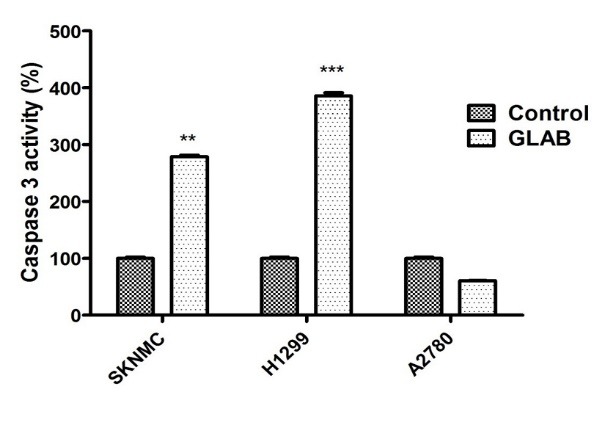
The effect of IC50 concentration of Glab on the caspase-3 activity of cancerous cells. Caspase-3 activity was measured by colorimetric detection and expressed as percent of control. Data are expressed as the mean± S.E.M of three separate experiments. ** *P* < 0.01, *** *P* <0.001 vs. control.

The results of the cell proliferation assay demonstrated the potential of Glab in inhibiting the proliferation of cancer cells, and the exposure of different cells to this herbal compound significantly caused to induce the death in cells. To understand the mechanism of the death (apoptosis or necrosis) induced by Glab, the key parameters involved in the apoptosis were investigated. Caspase-3 activity is one of the key markers of cell apoptosis, and activation of caspases is crucial in starting the apoptosis cascades. Figure 2 exhibits the capase-3 activity of different groups of cells before and after exposure to the IC_50_ concentration of Glab. It is obvious that for H1299 and SKNMC cells, the amounts of cleaved caspase-3 increased significantly upon the application of Glab, confirming that this herbal compound could activate apoptosis in the performed cancerous cells. This compound possessed the most significant effect on H1299 cells compared to other cell lines. Glab caused 3.72 fold increase in caspase-3 activity, while this value was 2.84 fold for SKNMC cells. The caspase-3 activity did not increase after exposure to Glab in A2780 cells.

### Effect of Glab on the mitochondrial membrane potential of cancerous calls

**Figure 3:**
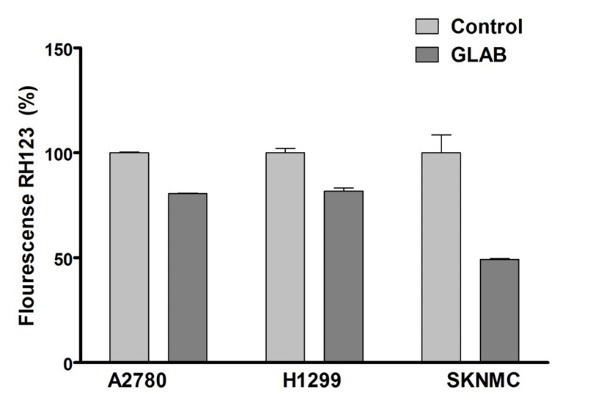
The effect of IC50 concentration of Glab on mitochondrial membrane potential (MMP) collapse as detected by Rhodamine 123. Data are expressed as the mean± SEM of three separate experiments. ** *P* <0.01 vs. control.

To further elucidate the mechanisms by which Glab induced the cell death in cancerous cell lines, we examined another key marker of the apoptosis. The mitochondria play a crucial role in activating apoptosis in mammalian cells. To elucidate whether application of Glab affects permeabilization of the mitochondrial membrane and consequently induces the cell death, the MMP was determined before and after exposure to the IC_50_ concentration of Glab (Figure 3). It was observed that Glab in its IC_50_ concentration considerably reduced the MMP of H1299 cells. Approximately 50 % decrease in rhodamin 123 florescence was observed in H1299 groups.

### Effect of Glab on regulating Bcl2 and Bax gene expression

**Figure 4:**
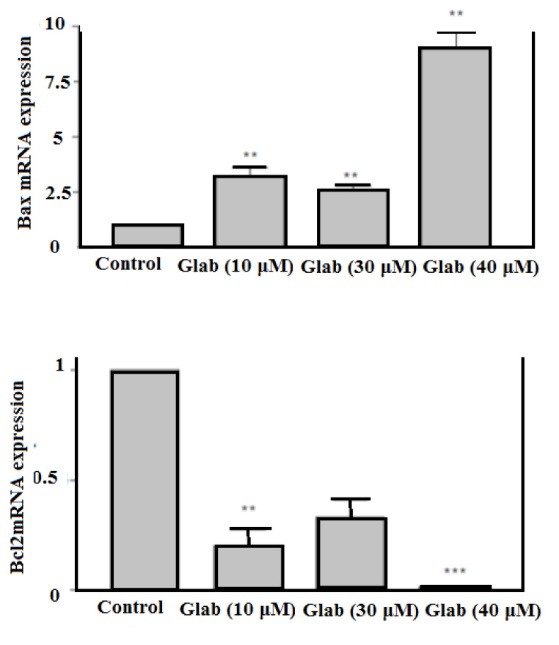
The effect of Glab on Bax and Bcl-2 gene expressions of H1299 Cells. Data are expressed as the mean± SEM of three separate experiments. ** *P* < 0.01, *** *P* < 0.001 vs. control.

The effect of Glab (10-40 μM) on the expression of gens participating in the mitochondrial apoptotic pathway (Bcl2 and Bax) was examined on the H1299 cell line using the RT-PCR method (Figure 4). These cells were selected based on the results of the MMP test, indicating that Glab possessed the most significant effect on the MMP of H1299 cells. The results revealed the fact that the obtained phytochemical could successfully up-regulate the pro-apoptotic Bax genes and down-regulate the anti-apoptotic Bcl2 gene expression via a dose-dependent manner. Among the performed doses of Glab, the highest concentration (40 μM) possessed the most significant effect on the regulation of these apoptotic gene expressions.

### Effect of Glab on the DOX-induced cytotoxicity in cancerous cells

**Figure 5:**
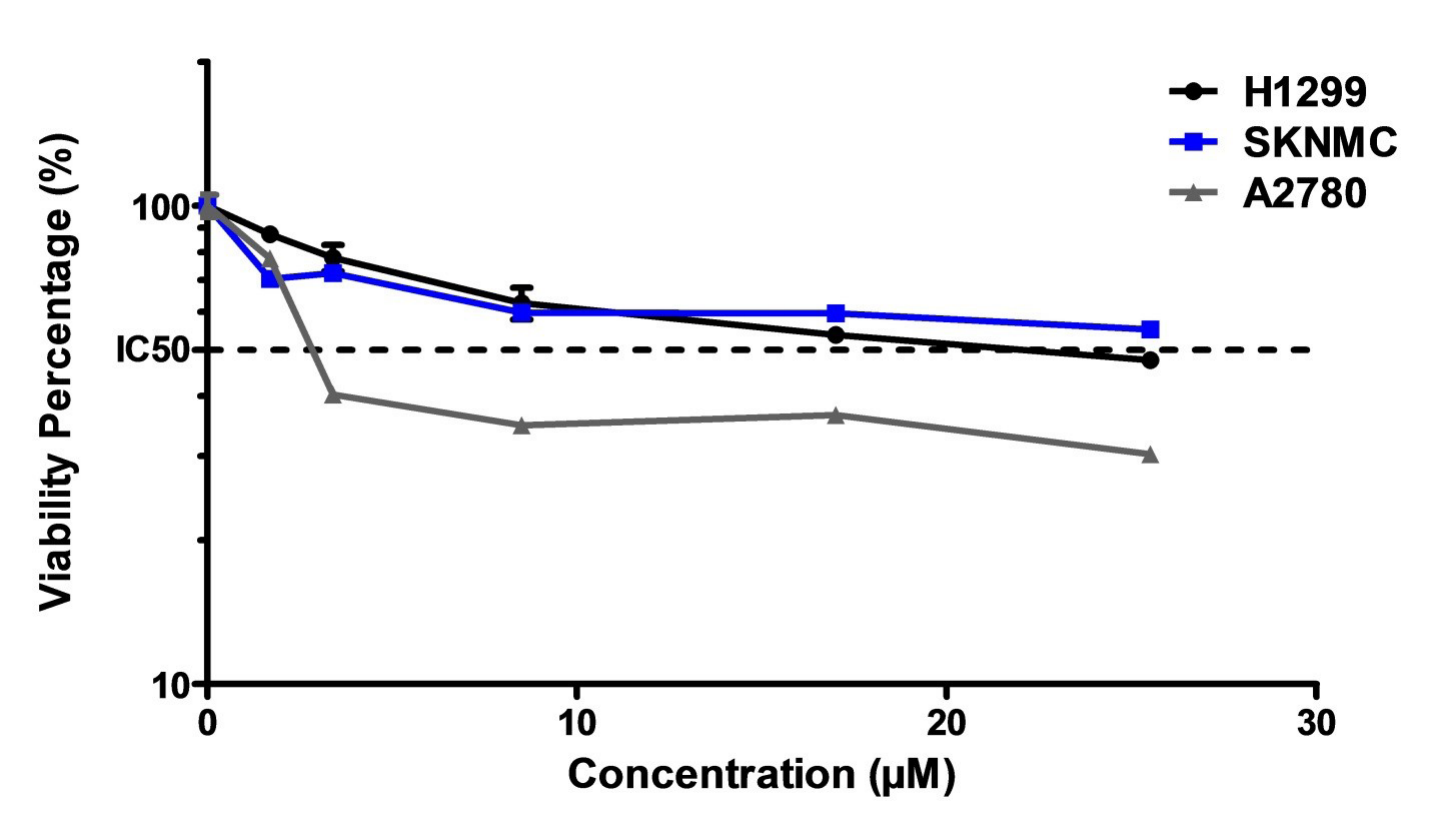
The effect of DOX on the cell viability of different cancerous cells. The cell viability was determined by MTT assay after 24 h exposure as described in materials and methods. Data are expressed as the mean± SEM of three separate experiments.

**Figure 6:**
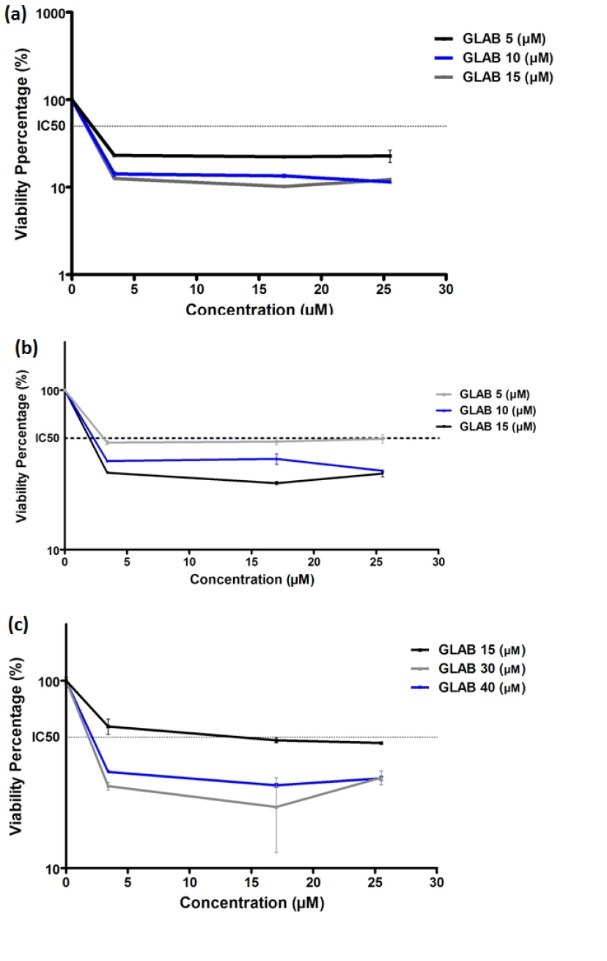
The effect of different concentrations of Glab on the DOX-induced toxicity toward (a) A2780, (b) SKNMC, and (c) H1299 cells. The cell viability was determined by MTT assay after 24 h exposure as described in materials and methods. Data are expressed as the mean±SEM of three separate experiments.

The aforementioned results of previous section confirmed that Glab could be a promising anticancer therapeutic, which stimulated the authors to investigate its possibility for co-administration with anticancer drugs such as DOX, to examine whether this compound can potentiate the cytotoxicity of DOX toward cancerous cells. In order to achieve this goal, firstly the cytotoxicity of DOX alone was investigated on each cell line separately. Figure 5 illustrates the effect of different concentrations of DOX on the viability of these cells in comparison to the control group (viability 100%). It could be observed that DOX could inhibit the proliferation of all the cancerous cells and caused significant cytotoxic effect toward these cells. For all the three cell lines, IC_50_ could be achieved and the corresponding IC_50_ values of DOX toward H1299, SKNMC, and A2780 were 28.9, 22.7 and 4.1 μM, respectively. This indicates that the most significant cytotoxic potential of DOX could be observed against ovarian carcinoma cells, in which up to 50% of cells were killed after exposure of 4.1 µM of DOX. Next, the viability percentage of cancerous cells in presence of DOX alone (Figure 5) was compared to that of corresponding cells in presence of both Glab (5, 10, and 15 μM) and DOX (Figure 6a-c). The results implied that exposure of all the three cell lines with Glab causes potentiating the cytotoxic activity of DOX and decreasing the cell viability. Although increasing the Glab concentration caused decreasing the IC_50_ toward each cancerous cell line, there was no significant difference between the IC_50_ value of samples with Glab 10 μM and those corresponding with Glab 15 μM; So Glab 10 μM was the most appropriate dosage for potentiating the anti-proliferation activity of DOX against cancerous cells. In comparison to the cells treated only with DOX, exposure of DOX-treated cancerous cells with Glab 10 μM caused 50, 90.9 and 89.28% reduction in IC_50_ against A2780, SKNMC, and H1299 cells, respectively.

### Effect of Glab on the accumulation of DOX in the H1299 cell line

**Figure 7:**
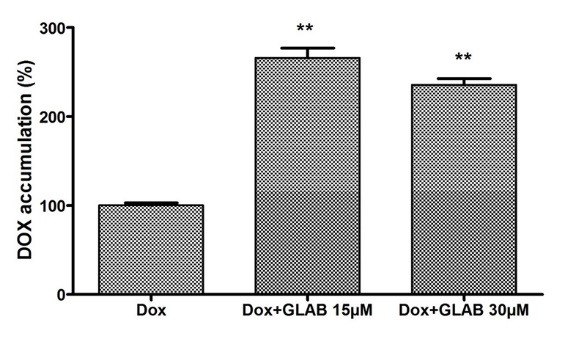
The effect of Glab (15 and 30 µM) on the accumulation of DOX (28 µM) in H1299 cells. Data are expressed as the mean± SEM of three separate experiments. ** *P*<0.01 vs. control.

According to the results of previous section, the most significant inhibitory potential of DOX was observed toward H1299 cells. This cell line was selected to elucidate the effect of Glab on the accumulation of DOX in cancerous cells. The accumulation of DOX (at the IC_50_ concentration of 28 μM) in the H1299, in presence and absence of Glab (15 and 30 μM) was examined and the results are demonstrated in Figure 7. It could be observed that Glab considerably increased the accumulation of DOX in these cells in a dose-dependent manner. The accumulation of DOX in these cells increased 2.8 and 2.5 times in presence of DOX 15 and 30 μM, respectively. Glab 15 μM possessed the most stimulatory effect on the accumulation of DOX in H1299 cells.

## Discussion

Cancer, as one of the major causes of human suffering and death, has increasingly prevailed in developing and developed countries. Cell death is considered a vital mechanism for tissue homeostasis and development, and the failure of cell death contributes to incidence of cancer. The programmed cell death, apoptosis, is a major therapeutic tactic in chemotherapy.^21,22^ Cancer chemotherapeutics are designed to kill cancerous cells via apoptosis induction or incapacitation of them; therefore, the modulation of apoptosis can be critical in chemoprevention and chemotherapy.^23^ Natural products are potent therapeutics in modulating the apoptosis and are new opportunities for cancer treatment and prevention.^24,25^ *G. glabra* and its active constituents have been extensively used as anti-proliferative agents and possess the ability to induce apoptosis in multiple types of cancerous cells.^26-29^ Glabridin, the main constituent of the *G. glabra* roots, promisingly exhibited protective and inhibitory effects against breast and liver cancers and^30-32^ hepatocellular carcinoma,^33^ by apoptosis induction in cancerous cells. In the present study, the anticancer and anti-apoptotic activity of Glab has been investigated against three different cancerous cell lines, corresponding to neuroblastma, lung and ovarian carcinoma. The cytotoxic effect of Glab against the cancerous cells corroborated the antiproliferative activity (up to 90% inhibitory) of this phytochemical at doses up to 75 µM. The apoptosis cascades may occur through two distinct cascades, in intrinsic and extrinsic. The MMP results in association with the caspase-3 activity results can be interpreted to find the possible pathway for apoptosis inhibition. Caspase-3 acts as an apoptotic executor via two pathways: the death receptor pathway and the mitochondrial pathway.^34^ In the intrinsic pathway, mitochondria possess both of the regulatory proteins, pro-apoptotic (including Bax) and anti-apoptotic (including Bcl2) ones, which play the major role in maintaining the MMP.^35,36^ In this study, we investigated which apoptotic pathway was involved in the anti-proliferation effect of Glab (35 µM) in different cancerous cells. According to the results, the amounts of cleaved caspase-3 of H1299 and SKNMC cells increased significantly upon the application of Glab, demonstrating that Glab activates apoptosis in these cell lines. On the contrary, the caspase-3 activity did not increase after exposure of Glab to A2780 cells. In addition, Glab significantly diminished the MMP in H1299 cell line. Therefore, it could be concluded that Glab induced apoptosis through intrinsic mitochondrial pathway in H1299 and SKNMC cells, while the apoptosis mechanism for A2780 cells could be probably through the extrinsic pathway. According to the previous studies into the anti-apoptotic activity of Glab, this phytochemical exerts apoptosis by inducing the caspase-3 activity in HL60 and Huh7 cells.^37,38^ It has been well-established that chemopreventive therapeutics induce the apoptosis in tumor cells in direct relation to their potential to reduce the anti-apoptotic proteins expression (Bcl-2 and Bcl-xL), and up-regulate the apoptotic promoter ones (Bax and Bak), eliciting the release of cytochrome c.^39^ In the present study, it was observed that Glab down-regulated the anti-apoptotic protein Bcl-2 level and prompted the apoptosis via elevating the Bax expression in H1299 cells, in a dose-dependent manner. This further confirmed that Glab stimulated apoptosis in these cells through a mitochondrion-mediated pathway. While, previous studies demonstrated that Glab could not alter the level of Bax and Bcl2 proteins in Huh7 cells. The studies also revealed that other anti-apoptotic and pro-apoptotic proteins were involved in the apoptosis mechanism of Glab toward Huh7 cells.^38^ Glab was also able to induce apoptosis in HL60 through up-regulation of the Bax and down-regulation of Bid proteins.^37^ According to the results, the considerable potential of Glab in inhibition of the proliferation and toxicity of the cancerous cells corresponding to the lung, neuroblastoma, and ovarian carcinoma, could be concluded in this study.

Although in a study by Tsai et al,^40^ exposure of A549, human non–small cell lung cancer, with Glab did not cause significant alteration in the viability percentage of cells, the treatment doses was lower (0-10 µM) than the doses performed in the present study. Despite the non-significant anti-proliferative effect of Glab toward A549 cells, it successfully affected the migration ability of the cells as well as their invasion ability, leading to metastasis inhibition of these cancerous cells. The anti-angiogenesis ability of Glab has also been proven in their study.

This study was the first report on the effect of Glab on the ovarian carcinoma; however, other studies have proven the effect of this isoflavone on the treatment of different ovarian syndromes. In a clinical study into the therapeutic effect of Glab on 32 women suffering from polycystic ovary syndrome, the administration of Glab (10 µM) ameliorated the syndrome by diminishing the serum testosterone and elevating the sex hormone-binding globulin level. Furthermore, all the patients reverted to regular menstrual cycles after 12-month treatment.^41^ Glab also exhibited cytotoxic and estrogenic and anti-proliferative activity toward human breast cancer cells.^42^ Licorice and its other isolates, including 18-β glycyrrhetinic acid exhibited anti-proliferative effect on human epithelial ovarian carcinoma cells, leading to a decreased Bcl-2 level and up-regulated cytosolic and mitochondrial Bax protein expression, and activated caspase-3.^43-45^

In addition to the apoptotic activity of Glab in the performed cancerous cells, in the present study, the effect of Glab on the cytotoxicity mediated by DOX in these three cancerous cells and its accumulation in the H1299 cell line was elucidated. The co-administration of anticancer drugs could exhibit a synergistic toxicity toward tumor cells and decrease damage of the normal cells. The combined treatment of all the three cancerous cells with DOX (IC_50_ concentration) and Glab (5-15 µM) potentiated the cytotoxicity against these cells and enhanced the intracellular accumulation of DOX in H1299 cells in a dose-dependent manner, demonstrating the beneficial effect of Glab to improve the efficacy of anti-cancer agents like DOX and to enhance the sensitivity of these cells to DOX. Glab has also been performed to improve the accumulation of DOX and to reduce the resistance of human carcinoma KB-C2 cells to this chemopreventive drug.^18^ This phytochemical could be considered a promising prescription medicament in combination with anti-cancer drugs like DOX.

## Conclusion

These results all confirmed the significant ability of Glab to be used as promising anti-cancer phytochemical against the lung cancer, ovarian carcinoma, and neuroblastoma, and its beneficial role in reducing the resistance of cancerous cells against anti-cancers like DOX, as well as prompting the cytotoxicity and efficacy of this anti-cancer drug.

## Ethical Issues

Not applicable.

## Conflict of Interest

The authors declared no conflict of interest for this study.

## Acknowledgments

The authors gratefully acknowledge the Research Concil of Kermanshah University of Medical Sciences for the financial support.
